# Regional and demographic variations in diabetes mellitus and myocardial infarction mortality among US adults: A retrospective observational analysis from 1999 to 2023

**DOI:** 10.1016/j.ijcrp.2025.200456

**Published:** 2025-06-18

**Authors:** Ahlam Safdar Hussain, Sumantra Kumar De, Griff Chatwin, Maryam Shahzad, Eeshal Zulfiqar, Vaisnavy Govindasamy, Megha Goel, Muhammad Muneeb Arshad, Mohib Naseer, Syed Rizwan Bokhari, Muhammad Atif Rauf, Sundas Hasan, Mushood Ahmed, Raheel Ahmed

**Affiliations:** a Fatima Memorial Hospital, Lahore, Pakistan; b Northwick Park Hospital, London, UK; c University Hospitals Birmingham NHS Foundation Trust, UK; d Dow University of Health Sciences, Karachi, Pakistan; e James Cook University Hospital, Middlesbrough, UK; f James Cook University Hospital, South Tees Hospitals NHS Foundation Trust, UK; g University of Rochester, NY, USA; h Department of Cardiac Electrophysiology AFIC & NIHD, Rawalpindi, Pakistan; i University Hospitals Leicester, UK; j Rawalpindi Medical University, Rawalpindi, Pakistan; k Royal Brompton Hospital, London, UK; l National Heart & Lung Institute, Imperial College London, UK

**Keywords:** Diabetes mellitus, Myocardial infarction, CDC WONDER, USA

## Abstract

**Background:**

Individuals with diabetes mellitus (DM) are at an increased risk of having myocardial infarction (MI). We aim to identify the trends in the mortality rates from DM and MI among US adults stratified by demographic and geographical parameters.

**Methods:**

The CDC-WONDER database was used to extract death certificate data for adults aged ≥25 years. Crude mortality rates (CMR) and age-adjusted mortality rates (AAMRs) per 100,000 persons were calculated, and temporal trends were identified by calculating annual percent change (APC) using JoinPoint regression analysis.

**Results:**

From 1999 to 2023, a total of 712,921 DM and MI-related deaths were reported among adults in the United States. The AAMR significantly declined from 18.99 in 1999 to 10.20 in 2018 Following this, the AAMR rose to 12.27 in 2021, with an APC of 7.2 (95 % CI: 4.7 to 8.9). This was followed by a decline in AAMR to the pre-pandemic levels (AAMR: 9.6 in 2023). The AAMR for males was considerably higher compared to women throughout the study period (16.5 vs. 10.2). Non-Hispanic Black or African American people had the highest AAMR (19.5), followed by Hispanic or Latino people (15.3), NH white people (12.5), and NH other populations (11.2). Southern region and rural areas experienced higher mortality rates compared to urban areas.

**Conclusion:**

We observed decreasing trends in DM and myocardial infarction-related deaths throughout the study period, however, a surge was noted during the COVID-19 pandemic. Mortality is higher among men, NH black populations, and rural areas.

## 1 Introduction

Diabetes mellitus (DM) is emerging as one of the most significant health threats in the twenty-first century. By 2030, it is projected that approximately 360 million individuals globally will be affected [1,2]. The primary causes of death in individuals with DM are largely attributed to coronary artery disease, along with elevated risks of stroke and peripheral vascular disease, known as macrovascular complications [3]. Notably, a significant proportion of deaths in individuals with diabetes are linked to atherothrombotic events and their consequences [[3], [4], [5]].

Individuals with DM face a three-fold increased risk of acute myocardial infarction (AMI) compared to those without DM, with AMI typically occurring 15 years earlier [6]. MI is one of the leading causes of death in individuals with DM. DM patients without a history of coronary artery disease (CAD) face the same risk of major coronary events as those with CAD, with over 20 % risk of a first MI within 10 years. Furthermore, the risk of MI recurrence in DM patients with a history of MI exceeds 40 % [[6], [7], [8], [9]]. Not only is diabetes a common comorbidity in MI patients, but it also significantly raises the risk of morbidity, mortality, and recurrence of MI compared to non-diabetic individuals [10]. This heightened risk is evident both in the acute phase of MI and in the years following the event. Although prior studies have examined mortality trends in diabetes and MI, further analysis is warranted to assess more recent patterns and disparities in the United States. This study utilizes the Centers for Disease Control and Prevention's Wide-ranging Online Data for Epidemiologic Research (CDC WONDER) database to examine mortality trends from 1999 to 2023, with a focus on demographic and regional differences in the United States.

## 2 Methods

### 2.1 Study setting and population

The CDC WONDER database was used to extract deaths related to DM and MI that occurred in the United States. CDC-WONDER is a comprehensive database of death certificate data from the fifty states of the USA as well as the District of Columbia. The Multiple Cause-of-Death Public Use record death certificates were studied to identify records in which DM and MI were mentioned as multiple cause of death on nationwide death certificates. This database has previously been used to determine trends in mortality of DM and MI. Patients were identified using the International Classification of Diseases 10th Revision Clinical Modification (ICD-10-CM) codes E10-E14 for DM and I21-I22 for MI in individuals ≥25 years of age [11,12]. This study did not require Institutional review board approval, as we used a publicly available, de-identified dataset provided by the government. The study adheres to the reporting standards outlined in the Strengthening the Reporting of Observational Studies in Epidemiology (STROBE) guidelines [13].

### 2.2 Data abstraction

Data on DM and MI-related deaths and population sizes were extracted. Demographics (sex, race/ethnicity, and age), and regional information (urban-rural and state) were extracted from 1999 to 2023. Race/ethnicities were delineated as non-Hispanic (NH) white, NH Black or African American, NH others (NH Asian or Pacific Islander, NH American Indian or Alaska Native, etc), and Hispanics or Latinos. These race/ethnicity categories have previously been used within analyses from the CDC WONDER database and rely on reported data on death certificates. For age stratification, age was divided into the following categories: 25–34, 35–44, 45–54, 55–64, 65–74, 75–84, and 85 years and older. Trends in mortality from DM and MI were evaluated based on state-specific variations, U.S. census regions (Northeast, Midwest, South, West), and county-level urbanization classifications. Counties were categorized as rural (micropolitan, noncore regions) or urban (large central metro, large fringe metro, medium metro, small metro) based on the 2013 National Center for Health Statistics Urban-Rural Classification Scheme [14].

### 2.3 Statistical analysis

Crude and age-adjusted mortality rates per 100,000 population were determined. Crude mortality rates (CMRs) were determined by dividing the number of DM and MI-related deaths by the corresponding US population of that year. Age adjusted mortality rates (AAMRs) were calculated by standardizing the DM and MI-related deaths to the 2000 US population as previously described [15]. The Joinpoint Regression Program (Joinpoint V 5.1.0.0, National Cancer Institute) was used to determine trends in AAMRs and CMRs using annual percent change (APC) [16]. This method identifies significant changes in AAMRs and CMRs over time by fitting log-linear regression models where temporal variation occurred. APCs with 95 % confidence intervals (CI) for the AAMRs and CMRs were calculated at the identified line segments linking join points using the Monte Carlo permutation test. APCs were considered increasing or decreasing if the slope describing the change in mortality was significantly different from zero using 2-tailed t testing. Statistical significance was set at p < 0.05.

## 3 Results

### 3.1 Overall

**Fig. 1:**
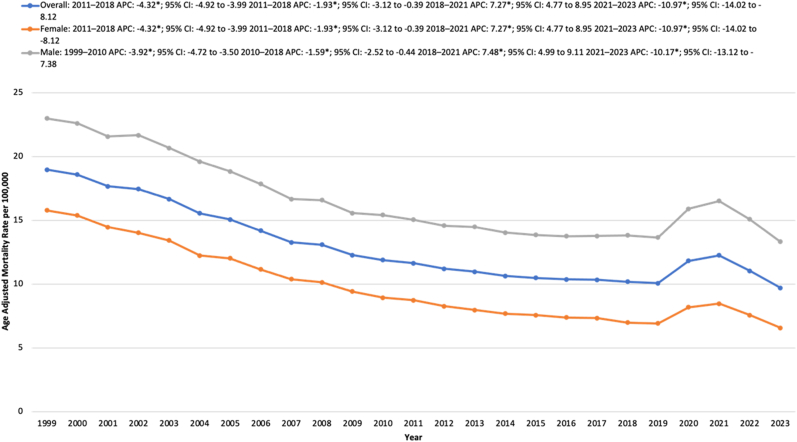
Overall and sex-stratified age-adjusted mortality rates (AAMRs) per 100,000 individuals in the United States, 1999 to 2023.

From 1999 to 2023, a total of 712,921 DM and MI-related deaths were reported among adults in the United States. The AAMR significantly declined from 18.99 in 1999 to 11.66 in 2011, with an APC of −4.32∗ (95 % CI: 4.92 to −3.99; p < 0.000001). The AAMR further decreased to 10.20 in 2018, reflecting an APC of −1.93∗ (95 % CI: 3.12 to −0.39; p = 0.027). Following this, the AAMR rose significantly to 12.27 in 2021, with an APC of 7.27∗ (95 % CI: 4.77 to 8.95; p < 0.000001). From 2021 to 2023, the AAMR again saw a decline from 12.27 to 9.72, with an APC of −10.97 (95 % CI: 14.02 to −8.12; p < 0.000001) (Table S1, Table S2, Fig. 1).

### 3.2 DM and MI-related mortality trends stratified by gender

From 1999 to 2023, males averaged a considerably higher AAMR than females. For males, the AAMR declined significantly from 23.36 in 1999 to 15.70 in 2010, with an APC of −3.92∗ (95 % CI: 4.72 to −3.50; p < 0.000001). This was followed by a further decrease to 14.05 in 2018, with an APC of −1.59∗ (95 % CI: 2.52 to −0.44; p = 0.018). The AAMR then rose sharply to 16.78 in 2021, reflecting an APC of 7.48∗ (95 % CI: 4.99 to 9.12; p < 0.000001). Following this peak, the AAMR again declined to 13.55 in 2023, with an APC of −10.17∗ (95 % CI: 13.12 to −7.38; p < 0.000001).

Similarly, the AAMR for females declined from 15.81 in 1999 to 8.29 in 2012, with an APC of −5.06∗ (95 % CI: 5.82 to −4.77; p < 0.000001). The AAMR further decreased to 7.01 in 2018 with an APC of −2.73∗ (95 % CI: 4.35 to −0.56; p = 0.019). From 2018 to 2021, the AAMR increased significantly from 7.01 to 8.49, reflecting an APC of 6.96∗ (95 % CI: 4.08 to 8.94; p < 0.000001). Following this peak, the AAMR significantly declined to 6.59 in 2023, with an APC of −11.78∗ (95 % CI: 15.40 to −8.42; p < 0.000001) (Table S2, Table S3, Fig. 1).

### 3.3 DM and MI-related mortality trends stratified by race

Throughout the study period, NH Black or African American individuals had the highest average AAMR, followed by Hispanic or Latino individuals, NH White individuals, and lastly NH Other populations.

For NH Black individuals, the AAMR remained stable from 1999 to 2003. The AAMR then declined significantly from 27.60 in 2003 to 16.27 in 2012, with an APC of −5.54∗ (95 % CI: 8.44 to −5.01; p < 0.000001). This was followed by a further decrease in AAMR to 14.33 in 2018, with an APC of −2.38∗ (95 % CI: 4.20 to −0.10; p = 0.042). From 2018 to 2021 the AAMR rapidly increased from 14.33 to 17.63, reflecting an APC of 7.85∗ (95 % CI: 4.54 to 10.19; p < 0.000001). The AAMR then declined to 13.85 in 2023, with an APC of −12.36∗ (95 % CI: 16.03 to −8.61; p < 0.000001).

For Hispanic or Latino individuals, the AAMR significantly declined from 25.96 in 1999 to 11.92 in 2017, reflecting an APC of −4.63∗ (95 % CI: 5.43 to −4.02; p = 0.0012). The AAMR then sharply rose to 15.79 in 2020, with an APC of 12.43∗ (95 % CI: 4.69 to 16.54; p = 0.010). Following this peak, the AAMR decreased to 10.99 in 2023 with an APC of −9.34∗ (95 % CI: 17.31 to −5.02; p = 0.0088).

Similarly for NH White individuals, the AAMR decreased from 17.50 in 1999 to 11.12 in 2010, with an APC of −4.29∗ (95 % CI: 4.83 to −3.96; p < 0.000001). The AAMR further declined to 9.67 in 2018 with an APC of −2.02 (95 % CI: 2.77 to −1.08; p = 0.0028). The AAMR then increased significantly to 11.54 in 2021, reflecting an APC of 6.99∗ (95 % CI: 4.77 to 8.47; p < 0.000001). This was followed by a decrease in AAMR to 9.16 in 2023 with an APC of −10.03∗ (95 % CI: 12.76 to −7.43; p < 0.000001).

**Fig. 2:**
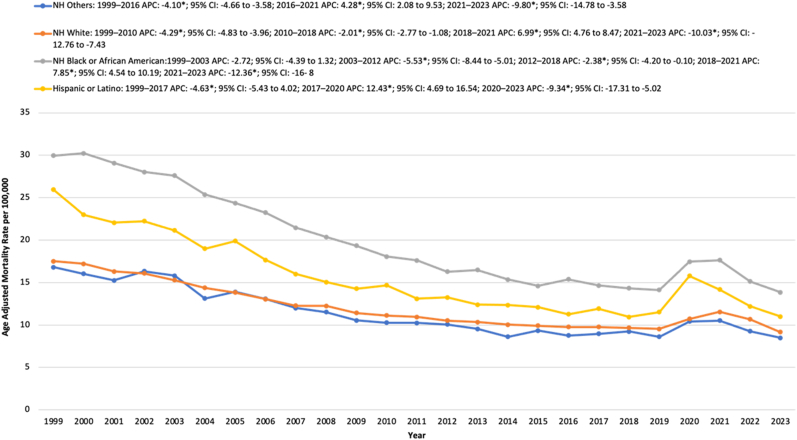
Age-adjusted mortality rates (AAMRs) per 100,000 individuals stratified by race/ethnicity in the United States, 1999 to 2023.

For NH Other populations, the AAMR significantly declined from 16.80 in 1999 to 8.77 in 2016, with an APC of −4.10∗ (95 % CI: 4.66 to −3.58; p = 0.0008). From 2016 to 2021, the AAMR increased from 8.77 to 10.51 with an APC of 4.28∗ (95 % CI: 2.08 to 9.53; p = 0052). This was followed by a decline in AAMR to 8.5 in 2023, reflecting an APC of −9.80∗ (95 % CI: 14.78 to −3.59; p < 0.000001). (Table S2, Table S4, Fig. 2).

### 3.4 DM and MI-related mortality trends stratified by geographical location

#### 3.4.1 Census region

From 1999 to 2023, the Southern region had the highest average AAMR, followed by the Midwest, West, and Northeast.

The Southern region saw a significant decline in AAMR from 19.07 in 1999 to 11.43 in 2013, reflecting an APC of −3.99∗ (95 % CI: 4.57 to −3.67; p = 0.0004). The AAMR remained relatively stable from 2013 to 2018. The AAMR then increased significantly from 11.12 in 2018 to 14.28 in 2021, with an APC of 9.27∗ (95 % CI: 6.49 to 11.39; p < 0.000001). This was followed by a significant decrease in AAMR to 11.50 in 2023, with an APC of −10.26∗ (95 % CI: 13.42 to −7.17; p < 0.000001).

In the Midwest region, the AAMR significantly decreased from 20.79 in 1999 to 13.06 in 2009, with an APC of −4.48∗ (95 % CI: 6.03 to −4.01; p < 0.000001). The AAMR further decreased to 10.81 in 2018 with an APC of −2.43∗ (95 % CI: 3.39 to −1.13; p = 0.0044). This was followed by an increase in AAMR to 12.50 in 2021, reflecting an APC of 5.41∗ (95 % CI: 2.54 to 7.17; p < 0.000001). The AAMR then declined to 9.51 in 2023, with an APC of −11.94∗ (95 % CI: 15.79 to −8.19; p < 0.000001).

The Western region saw a decline in AAMR from 16.82 in 1999 to 10.84 in 2011, reflecting an APC of −3.72∗ (95 % CI: 5.64 to −3.27; p < 0.0060). The AAMR then remained stable from 2011 to 2018. This was followed by an increase in AAMR from 9.84 in 2018 to 11.71 in 2021, with an APC of 6.51∗ (95 % CI: 3.40 to 8.65; p < 0.000001). The AAMR then decreased to 9.29 in 2023, with an APC of −10.63∗ (95 % CI: 14.36 to −6.63; p < 0.000001).

**Fig. 3:**
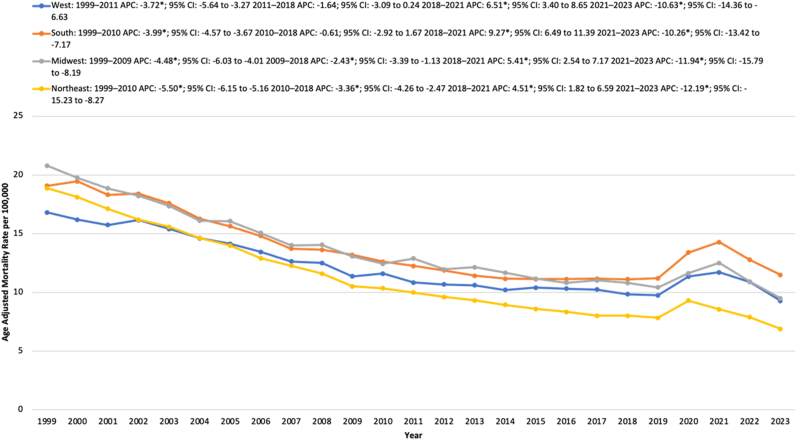
Age-adjusted mortality rates (AAMRs) per 100,000 individuals stratified by census region in the United States, 1999 to 2023.

Similarly, the Northeast region saw a significant decline in AAMR from 18.88 in 1999 to 10.36 in 2010, with an APC of −5.50∗ (95 % CI: 6.15 to −5.16; p < 0.000001). This was followed by a further decrease in AAMR to 8.02 in 2018 with an APC of −3.36∗ (95 % CI: 4.26 to −2.48; p = 0.0004). From 2018 to 2021, the AAMR increased from 8.02 to 8.56, reflecting an APC of 4.51∗ (95 % CI: 1.82 to 6.59; p = 0.0016). Following this, the AAMR declined to 6.89 in 2023, with an APC of −12.19∗ (95 % CI: 15.23 to −8.28; p = 0.0004) (Table S5–6, Fig. 3).

#### 3.4.2 Statewide

From 1999 to 2020, the states in the top 90th percentile included Arkansas, Mississippi, West Virginia, Kentucky and lastly, Tennessee. Whereas that in the bottom 10th percentile included Nevadah, Alaska, Montana, Connecticut and lastly, Minnesota. From 2021 to 2023, the states in the top 90th percentile included Mississippi, Arkansas, Kentucky, South Dakota and lastly, Tennessee. The states in the bottom 10th percentile included Connecticut, Alaska, Massachusetts, Nevada and lastly, New Hampshire (Table S5).

#### 3.4.3 Urban-rural

From 1999 to 2020, rural areas had a considerably higher average AAMR than urban areas. For rural areas, the AAMR significantly declined from 21.78 in 1999 to 15.89 in 2010, with an APC of −2.99∗ (95 % CI: 5.80 to −1.27; p = 0.044). The AAMR remained stable from 2010 to 2018. From 2018 to 2020, the AAMR significantly increased from 15.52 to 18.04, reflecting an APC of 8.52∗ (95 % CI: 0.96 to 12.28; p = 0.017).

**Fig. 4:**
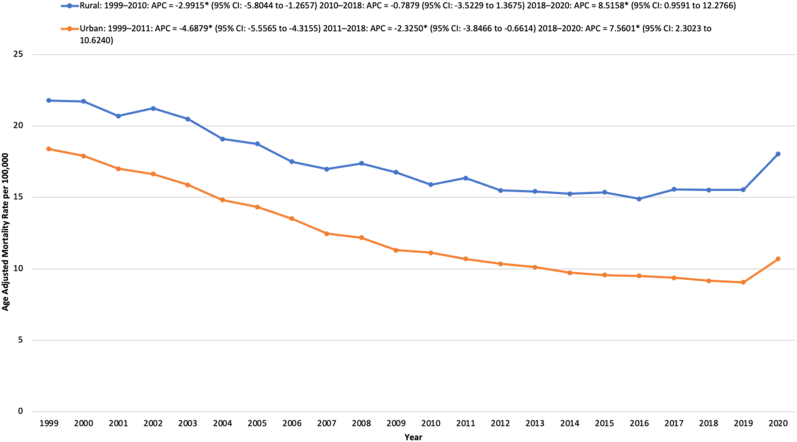
Age-adjusted mortality rates (AAMRs) per 100,000 individuals stratified by urbanization in the United States, 1999 to 2020. **∗** Data for urbanization AAMRs was unavailable for 2021-2023

The urban areas saw a significant decline in AAMR from 18.39 in 1999 to 10.70 in 2011, with an APC of −4.69∗ (95 % CI: 5.56 to −4.32; p = 0.0004). The AAMR further decreased to 9.16 in 2018, reflecting an APC of −2.33∗ (95 % CI: 3.85 to −0.66; p = 0.016). From 2018 to 2020, the urban areas saw a significant rise in AAMR from 9.16 to 10.69, reflecting an APC of 7.56∗ (95 % CI: 2.30 to 10.62; p = 0.0056). (Table S7, Fig. 4).

### 3.5 DM and MI-related mortality trends stratified by 10- year age groups

In the analysis stratified by age groups, the highest CMRs were observed in the 85+ and 75–84 age categories, followed by the 65–74, 55–64, and 45–54 age groups. Conversely, the 35–44 and 25–34 age groups displayed the lowest CMRs (Table S8).

## 4 Discussion

This retrospective analysis shows that the overall AAMR for DM and MI-related deaths declined from 1999 to 2018, followed by a sharp rise during the COVID-19 pandemic. Gender and racial disparities were evident, with males and NH Black or African American populations experiencing higher AAMRs compared to females and other racial/ethnic groups. Moreover, regional and geographic disparities were observed, with the Southern region and rural areas displaying the highest mortality rates, in contrast to the Northeast and urban areas, where AAMRs were comparatively lower.

CVD including MI remains the leading cause of death among individuals with DM, compounded by the ongoing obesity epidemic and the increasing prevalence of hypertension, hyperlipidemia, obesity, smoking, and sedentary lifestyles [9,11,17]. Data from the National Health and Nutrition Examination Survey highlights suboptimal control of modifiable risk factors in diabetic patients, with significant proportions failing to meet target goals for low-density lipoprotein cholesterol, blood pressure, and HbA1c levels during the 1988–2010 period [18]. Moreover, the population-attributable risk of diabetes for MI has risen, emphasizing the potential urgent need for early and aggressive management of DM to help reduce MI-related mortality at the population level [11]. Our study provides an updated estimate of the MI and diabetes-related mortality burden, which can potentially play a in role guiding policy measures and identifying areas for targeted interventions.

A decrease in mortality rates was observed from 1999 to 2018 which can be attributed to the advancements in the management of diabetes [19]. Moreover, newer treatments like SGLT2 inhibitors, GLP-1 receptor agonists, and advancements in intravascular imaging show promise in reducing DM and CVD-related morbidity and mortality [8,[19], [20], [21], [22]]. The sharp rise in mortality rates from 2019 to 2021 is likely linked to the widespread effects of the COVID-19 pandemic. Individuals with pre-existing conditions like DM faced increased vulnerability to severe outcomes, including respiratory failure and cardiovascular complications, contributing to elevated death rates [[23], [24], [25]]. Furthermore, the pandemic strained healthcare systems globally, leading to significant disruptions in routine care, delayed diagnoses, and postponed treatments for chronic illnesses. Lockdowns and social restrictions brought about behavioral shifts, such as heightened stress levels, decreased physical activity, and poor dietary habits, which may have compounded the challenges of managing health conditions.

Interestingly, while CVD-related mortality has plateaued after years of decline, DM-related mortality has reversed course in the past decade as shown by recent studies [11,17]. This reversal is of particular public health concern, as diabetes not only exacerbates cardiovascular complications but also increases the risk of other non-cardiovascular complications. Effective population-level management of DM could, therefore, potentially have far-reaching positive effects on overall life expectancy. Furthermore, racial and ethnic disparities in DM and MI mortality persist, with NH Black adults experiencing high mortality rates compared to NH White adults. These disparities are likely attributed to socioeconomic disadvantages, limited access to healthcare, and a higher prevalence of nontraditional cardiovascular risk factors such as subclinical inflammation and dyslipidemia [[26], [27], [28]].

Geographic and urban-rural disparities in mortality are also prominent. Rural areas, which historically have higher all-cause mortality, continue to see worse outcomes in both DM and MI mortality rates compared to urban areas. This gap has expanded over the past two decades, with urban areas experiencing a decrease in mortality rates. These disparities are further influenced by state-specific factors, including healthcare policies, Medicaid expansion, and levels of healthcare expenditure, all of which might impact mortality rates at the state level [[29], [30], [31]]. Diabetes prevalence has risen sharply in several middle-income countries, such as Brazil and Portugal, highlighting the growing burden and shifting epidemiology of type 2 diabetes [[32], [33], [34]]. Additionally, recent guidelines emphasize the critical role of managing hypertension in diabetes care [35]. The COVID-19 pandemic further complicated mortality assessments, especially in individuals with chronic conditions like diabetes [36,37].

### 4.1 Limitations

This study has some limitations which need to be considered. The study relied on the CDC WONDER database, which derives mortality data primarily from death certificates. Misclassification is a concern, as deaths from conditions like DM or MI may have been inaccurately coded, potentially resulting in underestimation of the true disease burden. The database also lacks crucial clinical details, such as disease severity, lipid profile, sugar level, HbA1c levels, association of HTN, comorbidities, or the use of therapies, limiting the ability to address mortality patterns. Additionally, individual-level variables like socioeconomic status, healthcare access, and lifestyle factors are missing. While adjustments for population age structure were made, they may not fully account for temporal changes in healthcare practices or evolving risk factors. It is important to mention that while we focused on diabetes and MI related mortality, it is important to recognize that other factors including smoking, management of hypertension and dyslipidemia, rising obesity rates, and population ageing also contribute to cardiovascular mortality trends and may confound the associations observed.

## 5 Conclusion

Our study found that DM and MI mortality has decreased over the years in the US but significantly increased during the COVID-19 pandemic. The mortality is higher among men and NH Black individuals. Geographically, the highest burden was observed in the Southern United States and rural areas, emphasizing the urgent need for public health strategies to mitigate disparities and improve healthcare outcomes in this vulnerable population.

## CRediT authorship contribution statement

**Ahlam Safdar Hussain:** Writing – original draft, Project administration. **Sumantra Kumar De:** Writing – original draft. **Griff Chatwin:** Writing – original draft, Formal analysis, Data curation. **Maryam Shahzad:** Writing – original draft, Methodology, Formal analysis, Data curation. **Eeshal Zulfiqar:** Methodology, Formal analysis, Data curation. **Vaisnavy Govindasamy:** Writing – original draft. **Megha Goel:** Writing – original draft. **Muhammad Muneeb Arshad:** Writing – original draft, Methodology. **Mohib Naseer:** Writing – original draft. **Syed Rizwan Bokhari:** Project administration, Investigation. **Muhammad Atif Rauf:** Writing – original draft. **Sundas Hasan:** Writing – original draft. **Mushood Ahmed:** Writing – review & editing, Visualization, Validation, Supervision. **Raheel Ahmed:** Writing – review & editing, Visualization, Validation, Supervision.

## Ethical approval

No ethical approval was required for the study.

## Consent

No consent was needed.

## Data availability statement

All data generated or analyzed during this study are included in this article. Further inquiries can be directed to the corresponding author.

## Financial support

No financial support was received for the study.

## Declaration of competing interest

We attest that the article is the Authors' original work, has not received prior publication and is not under consideration for publication elsewhere. We adhere to the statement of ethical publishing.

On behalf of all Co-Authors, the corresponding Author shall bear full responsibility for the submission. Any changes to the list of authors, including changes in order, additions or removals will require the submission of a new author agreement form approved and signed by all the original and added submitting authors.

All authors are requested to disclose any actual or potential conflict of interest including any financial, personal or other relationships with other people or organizations within three years of beginning the submitted work that could inappropriately influence, or be perceived to influence, their work. If there are no conflicts of interest, the COI should read: “The authors report no relationships that could be construed as a conflict of interest”.
