# Supplementary material for: Regional and demographic variations in diabetes mellitus and myocardial infarction mortality among US adults: A retrospective observational analysis from 1999 to 2023
Source: Int J Cardiol Cardiovasc Risk Prev. 2025 Jun 18;26:200456. doi: 10.1016/j.ijcrp.2025.200456 (PMC12221463; doi:10.1016/j.ijcrp.2025.200456)
Supplement: Multimedia component 1 [file mmc1.docx]

**Supplemental File**

**Table S1:** DM and MI related Deaths stratified by Gender and Race in the United States; 1999-2023.

| **Absolute number of deaths** | | | | | | | |
| --- | --- | --- | --- | --- | --- | --- | --- |
| **Year** | **Overall** | **Male** | **Female** | **NH Black or African American** | **NH White** | **Hispanic or Latino** | **NH Others** |
| 1999 | 33604 | 17175 | 16429 | 4531 | 25850 | 2315 | 832 |
| 2000 | 33293 | 17070 | 16223 | 4623 | 25613 | 2131 | 837 |
| 2001 | 32178 | 16687 | 15491 | 4543 | 24539 | 2171 | 851 |
| 2002 | 32336 | 17112 | 15224 | 4486 | 24485 | 2329 | 951 |
| 2003 | 31463 | 16715 | 14748 | 4524 | 23573 | 2322 | 989 |
| 2004 | 29824 | 16183 | 13641 | 4230 | 22456 | 2208 | 872 |
| 2005 | 29539 | 15920 | 13619 | 4170 | 21942 | 2418 | 962 |
| 2006 | 28313 | 15480 | 12833 | 4075 | 20996 | 2248 | 950 |
| 2007 | 26982 | 14803 | 12179 | 3869 | 20008 | 2140 | 925 |
| 2008 | 27247 | 15134 | 12113 | 3776 | 20354 | 2143 | 923 |
| 2009 | 26021 | 14593 | 11428 | 3692 | 19248 | 2122 | 912 |
| 2010 | 25735 | 14732 | 11003 | 3539 | 18934 | 2290 | 912 |
| 2011 | 25838 | 14873 | 10965 | 3609 | 19011 | 2195 | 980 |
| 2012 | 25503 | 14811 | 10692 | 3416 | 18639 | 2339 | 1042 |
| 2013 | 25627 | 15159 | 10468 | 3578 | 18637 | 2318 | 1044 |
| 2014 | 25382 | 15073 | 10309 | 3491 | 18374 | 2447 | 996 |
| 2015 | 25738 | 15358 | 10380 | 3439 | 18493 | 2566 | 1164 |
| 2016 | 25908 | 15572 | 10336 | 3701 | 18479 | 2513 | 1135 |
| 2017 | 26423 | 15968 | 10455 | 3667 | 18688 | 2767 | 1224 |
| 2018 | 26677 | 16484 | 10193 | 3693 | 18906 | 2689 | 1332 |
| 2019 | 26844 | 16566 | 10278 | 3708 | 18895 | 2904 | 1283 |
| 2020 | 32024 | 19695 | 12329 | 4738 | 21443 | 4164 | 1626 |
| 2021 | 32893 | 20428 | 12465 | 4700 | 22529 | 3848 | 1603 |
| 2022 | 30603 | 19054 | 11549 | 4108 | 21365 | 3458 | 1493 |
| 2023 | 26926 | 16877 | 10049 | 3801 | 18434 | 3092 | 1383 |

**Table S2:** Average Annual Percentage (95% confidence interval) of DM and MI related Mortality in the United States; 1999-2023.

| **Variable** | **Subgroup** | **Time Period** | **APC (95% CI)** | **p-value** |
| --- | --- | --- | --- | --- |
| **Gender** |  |  |  |  |
|  | Overall | 2011–2018 | -4.32* (-4.92 to -3.99) | <0.000001 |
|  |  | 2018–2021 | 7.27* (4.77 to 8.95) | <0.000001 |
|  |  | 2021–2023 | -10.97* (-14.02 to -8.12) | <0.000001 |
|  | Female | 2012–2018 | -5.06* (-5.82 to -4.77) | <0.000001 |
|  |  | 2018–2021 | 6.96* (4.08 to 8.94) | <0.000001 |
|  |  | 2021–2023 | -11.78* (-15.40 to -8.42) | <0.000001 |
|  | Male | 1999–2010 | -3.92* (-4.72 to -3.50) | <0.000001 |
|  |  | 2018–2021 | 7.48* (4.99 to 9.11) | <0.000001 |
|  |  | 2021–2023 | -10.17* (-13.12 to -7.38) | <0.000001 |
| **Race/Ethnicity** | NH Black or African American | 1999–2001 | 16.86* (8.12 to 24.64) | <0.000001 |
|  |  | 2001–2010 | 0.80 (-0.65 to 2.12) | 0.173 |
|  |  | 2018–2021 | 12.17 (-2.14 to 14.87) | 0.079 |
|  | NH White | 1999–2001 | 22.33* (13.52 to 28.30) | <0.000001 |
|  |  | 2018–2021 | 13.12* (9.87 to 15.22) | 0.002 |
|  | Hispanic or Latino | 1999–2005 | 8.41* (3.89 to 16.59) | 0.024 |
|  |  | 2018–2021 | 9.16 (-0.38 to 11.63) | 0.062 |
| **Census Region** | Northeast | 1999–2010 | -5.50* (-6.15 to -5.16) | <0.000001 |
|  |  | 2018–2021 | 4.51* (1.82 to 6.59) | 0.0016 |
|  | South | 1999–2010 | -3.99* (-4.57 to -3.67) | 0.0004 |
|  |  | 2018–2021 | 9.27* (6.49 to 11.39) | <0.000001 |
| **Age Groups** | 25–34 years | 1999–2023 | -1.01* (-1.98 to -0.15) | 0.03 |
|  | 55–64 years | 1999–2009 | -4.62* (-5.61 to -4.05) | <0.000001 |
|  |  | 2018–2021 | 7.83* (4.84 to 9.61) | <0.000001 |
|  | 65–74 years | 1999–2010 | -5.1612* (-5.7618 to -4.7443) | <0.000001 |
|  |  | 2010–2018 | -1.8295* (-2.8451 to -0.8394) | 0.0056 |
|  |  | 2018–2021 | 5.9870* (3.4279 to 7.6431) | <0.000001 |
|  |  | 2021–2023 | -10.4529* (-13.3757 to -7.5338) | <0.000001 |
|  | 75–84 years | 1999–2012 | -4.5511* (-5.0461 to -4.2833) | <0.000001 |
|  |  | 2012–2018 | -2.0340* (-3.4627 to -0.2655) | 0.0360 |
|  |  | 2018–2021 | 7.2545* (4.7942 to 9.0383) | <0.000001 |
|  |  | 2021–2023 | -11.2760* (-14.0755 to -8.6173) | <0.000001 |
|  | 85+ years | 1999–2001 | 23.85* (13.39 to 31.29) | <0.000001 |
|  |  | 2001–2007 | 3.36* (1.40 to 6.52) | 0.0056 |
|  |  | 2007–2018 | 0.17 (-1.46 to 0.65) | 0.745 |
|  |  | 2018–2021 | 13.62* (10.05 to 16.00) | 0.0008 |
|  |  | 2021–2023 | -0.89 (-4.16 to 2.84) | 0.541 |

APC = Annual Percentage Change

CI = Confidence Interval

*indicates statistical significance (p < 0.05)

**Table S3:** Overall and Sex stratified DM and MI related Age Adjusted Mortality Rates per 100,000 in the United States; 1999-2023.

| **Age Adjusted Mortality Rate per 100,000 (95% CI)** | | | |
| --- | --- | --- | --- |
| **Year** | **Overall** | **Male** | **Female** |
| 1999 | 18.78 (19.19 - 0.1) | 23.01 (23.72 - 0.18) | 15.56 (16.05 - 0.12) |
| 2000 | 18.4 (18.8 - 0.1) | 22.62 (23.32 - 0.18) | 15.16 (15.64 - 0.12) |
| 2001 | 17.5 (17.88 - 0.1) | 21.58 (22.26 - 0.17) | 14.25 (14.7 - 0.12) |
| 2002 | 17.28 (17.67 - 0.1) | 21.68 (22.35 - 0.17) | 13.81 (14.26 - 0.11) |
| 2003 | 16.51 (16.88 - 0.09) | 20.68 (21.32 - 0.17) | 13.21 (13.64 - 0.11) |
| 2004 | 15.39 (15.74 - 0.09) | 19.62 (20.25 - 0.16) | 12.05 (12.47 - 0.11) |
| 2005 | 14.92 (15.26 - 0.09) | 18.85 (19.46 - 0.15) | 11.84 (12.24 - 0.1) |
| 2006 | 14.04 (14.37 - 0.08) | 17.87 (18.45 - 0.15) | 10.97 (11.36 - 0.1) |
| 2007 | 13.12 (13.44 - 0.08) | 16.69 (17.25 - 0.14) | 10.21 (10.59 - 0.1) |
| 2008 | 12.95 (13.26 - 0.08) | 16.61 (17.16 - 0.14) | 9.98 (10.34 - 0.09) |
| 2009 | 12.13 (12.43 - 0.08) | 15.59 (16.11 - 0.13) | 9.27 (9.62 - 0.09) |
| 2010 | 11.77 (12.06 - 0.07) | 15.44 (15.96 - 0.13) | 8.78 (9.12 - 0.09) |
| 2011 | 11.52 (11.81 - 0.07) | 15.07 (15.57 - 0.13) | 8.59 (8.92 - 0.08) |
| 2012 | 11.08 (11.36 - 0.07) | 14.61 (15.1 - 0.12) | 8.13 (8.45 - 0.08) |
| 2013 | 10.85 (11.13 - 0.07) | 14.51 (14.99 - 0.12) | 7.83 (8.14 - 0.08) |
| 2014 | 10.51 (10.78 - 0.07) | 14.05 (14.51 - 0.12) | 7.56 (7.86 - 0.08) |
| 2015 | 10.38 (10.64 - 0.07) | 13.87 (14.33 - 0.12) | 7.43 (7.73 - 0.08) |
| 2016 | 10.26 (10.52 - 0.07) | 13.77 (14.22 - 0.11) | 7.26 (7.55 - 0.07) |
| 2017 | 10.22 (10.47 - 0.06) | 13.79 (14.24 - 0.11) | 7.22 (7.51 - 0.07) |
| 2018 | 10.08 (10.33 - 0.06) | 13.84 (14.27 - 0.11) | 6.87 (7.15 - 0.07) |
| 2019 | 9.97 (10.22 - 0.06) | 13.67 (14.1 - 0.11) | 6.8 (7.08 - 0.07) |
| 2020 | 11.7 (11.96 - 0.07) | 15.92 (16.38 - 0.12) | 8.06 (8.36 - 0.08) |
| 2021 | 12.14 (12.41 - 0.07) | 16.54 (17.01 - 0.12) | 8.34 (8.64 - 0.08) |
| 2022 | 10.93 (11.18 - 0.06) | 15.11 (15.56 - 0.11) | 7.44 (7.72 - 0.07) |
| 2023 | 9.6 (9.84 - 0.06) | 13.34 (13.76 - 0.11) | 6.45 (6.72 - 0.07) |

**Table S4:** Race stratified DM and MI related Age Adjusted Mortality Rates per 100,000 in the United States; 1999-2023.

| **Age Adjusted Mortality Rate per 100,000 (95% CI)** | | | | |
| --- | --- | --- | --- | --- |
| **Year** | **NH Black or African American** | **NH White** | **Hispanic or Latino** | **NH Others** |
| 1999 | 29.95 (29.06 - 30.83) | 17.5 (17.29 - 17.71) | 25.96 (24.85 - 27.06) | 16.8 (15.6 - 18) |
| 2000 | 30.22 (29.34 - 31.1) | 17.22 (17.01 - 17.43) | 22.99 (21.97 - 24) | 16.03 (14.9 - 17.16) |
| 2001 | 29.07 (28.21 - 29.92) | 16.3 (16.1 - 16.51) | 22.07 (21.1 - 23.03) | 15.25 (14.18 - 16.32) |
| 2002 | 28.04 (27.21 - 28.87) | 16.07 (15.87 - 16.27) | 22.25 (21.31 - 23.2) | 16.33 (15.25 - 17.41) |
| 2003 | 27.6 (26.78 - 28.42) | 15.27 (15.07 - 15.46) | 21.15 (20.25 - 22.05) | 15.8 (14.78 - 16.83) |
| 2004 | 25.37 (24.59 - 26.15) | 14.39 (14.21 - 14.58) | 18.98 (18.15 - 19.8) | 13.12 (12.21 - 14.03) |
| 2005 | 24.36 (23.61 - 25.12) | 13.82 (13.64 - 14) | 19.9 (19.08 - 20.73) | 13.91 (13 - 14.83) |
| 2006 | 23.24 (22.51 - 23.97) | 13.06 (12.88 - 13.24) | 17.66 (16.9 - 18.42) | 13.07 (12.21 - 13.94) |
| 2007 | 21.46 (20.77 - 22.16) | 12.28 (12.11 - 12.45) | 16.01 (15.3 - 16.71) | 11.99 (11.19 - 12.79) |
| 2008 | 20.36 (19.7 - 21.03) | 12.26 (12.09 - 12.43) | 15.05 (14.38 - 15.71) | 11.53 (10.76 - 12.3) |
| 2009 | 19.35 (18.71 - 19.99) | 11.43 (11.27 - 11.59) | 14.27 (13.64 - 14.9) | 10.55 (9.84 - 11.25) |
| 2010 | 18.05 (17.44 - 18.66) | 11.12 (10.96 - 11.28) | 14.67 (14.04 - 15.29) | 10.28 (9.59 - 10.97) |
| 2011 | 17.61 (17.02 - 18.2) | 10.95 (10.79 - 11.11) | 13.1 (12.53 - 13.67) | 10.24 (9.58 - 10.9) |
| 2012 | 16.27 (15.71 - 16.84) | 10.52 (10.37 - 10.67) | 13.24 (12.69 - 13.8) | 10.08 (9.45 - 10.71) |
| 2013 | 16.49 (15.93 - 17.04) | 10.35 (10.19 - 10.5) | 12.41 (11.89 - 12.93) | 9.54 (8.95 - 10.14) |
| 2014 | 15.36 (14.83 - 15.88) | 10.04 (9.89 - 10.19) | 12.36 (11.85 - 12.87) | 8.62 (8.07 - 9.16) |
| 2015 | 14.61 (14.1 - 15.11) | 9.92 (9.78 - 10.07) | 12.1 (11.61 - 12.58) | 9.34 (8.79 - 9.89) |
| 2016 | 15.37 (14.86 - 15.88) | 9.78 (9.64 - 9.93) | 11.28 (10.82 - 11.74) | 8.77 (8.25 - 9.29) |
| 2017 | 14.65 (14.16 - 15.14) | 9.76 (9.61 - 9.9) | 11.92 (11.46 - 12.38) | 8.96 (8.45 - 9.47) |
| 2018 | 14.33 (13.85 - 14.81) | 9.67 (9.53 - 9.81) | 10.96 (10.53 - 11.39) | 9.25 (8.75 - 9.76) |
| 2019 | 14.13 (13.66 - 14.59) | 9.54 (9.4 - 9.68) | 11.52 (11.09 - 11.95) | 8.63 (8.15 - 9.11) |
| 2020 | 17.46 (16.95 - 17.97) | 10.72 (10.57 - 10.86) | 15.79 (15.3 - 16.29) | 10.42 (9.9 - 10.93) |
| 2021 | 17.63 (17.11 - 18.15) | 11.54 (11.38 - 11.69) | 14.19 (13.73 - 14.66) | 10.51 (9.99 - 11.03) |
| 2022 | 15.14 (14.67 - 15.62) | 10.67 (10.52 - 10.82) | 12.19 (11.77 - 12.61) | 9.26 (8.78 - 9.73) |
| 2023 | 13.85 (13.4 - 14.3) | 9.16 (9.03 - 9.3) | 10.99 (10.59 - 11.39) | 8.5 (8.05 - 8.96) |

**Table S5:** DM and MI related Age Adjusted Mortality Rates per 100,000 stratified by States in the United States; 1999-2023.

| **Age Adjusted Mortality Rate per 100,000 (95% CI)** | | |
| --- | --- | --- |
| **States** | **1999-2020** | **2021-2023** |
| Alabama | 10.32 (10.09 - 10.55) | 7.04 (6.57 - 7.51) |
| Alaska | 7.18 (6.51 - 7.85) | 5.23 (4.06 - 6.65) |
| Arizona | 8.41 (8.23 - 8.59) | 10.41 (9.95 - 10.88) |
| Arkansas | 22.76 (22.32 - 23.19) | 28.24 (27.01 - 29.46) |
| California | 14.33 (14.23 - 14.44) | 11.42 (11.2 - 11.64) |
| Colorado | 8.3 (8.08 - 8.52) | 7.65 (7.17 - 8.14) |
| Connecticut | 7.32 (7.11 - 7.54) | 3.82 (3.42 - 4.22) |
| Delaware | 12.59 (12.01 - 13.16) | 8.99 (7.88 - 10.1) |
| District of Columbia | 12.2 (11.45 - 12.95) | 10.75 (8.95 - 12.55) |
| Florida | 9.12 (9.02 - 9.21) | 9.34 (9.1 - 9.57) |
| Georgia | 9.28 (9.11 - 9.45) | 8.5 (8.12 - 8.87) |
| Hawaii | 10.27 (9.85 - 10.68) | 7.33 (6.49 - 8.17) |
| Idaho | 15 (14.5 - 15.51) | 15.94 (14.75 - 17.13) |
| Illinois | 13.54 (13.37 - 13.7) | 9.35 (9 - 9.69) |
| Indiana | 14.39 (14.16 - 14.63) | 13.94 (13.35 - 14.52) |
| Iowa | 13.32 (13.01 - 13.63) | 9.86 (9.16 - 10.55) |
| Kansas | 10.31 (10.01 - 10.61) | 8.89 (8.18 - 9.59) |
| Kentucky | 18.25 (17.93 - 18.57) | 23.62 (22.69 - 24.55) |
| Louisiana | 12.76 (12.49 - 13.03) | 11.25 (10.61 - 11.89) |
| Maine | 12.45 (12.01 - 12.89) | 11.75 (10.68 - 12.83) |
| Maryland | 14.63 (14.38 - 14.89) | 10.05 (9.54 - 10.56) |
| Massachusetts | 8.84 (8.66 - 9.02) | 5.29 (4.95 - 5.64) |
| Michigan | 12.88 (12.7 - 13.05) | 8.45 (8.09 - 8.81) |
| Minnesota | 8.13 (7.94 - 8.33) | 7.14 (6.69 - 7.58) |
| Mississippi | 21.35 (20.92 - 21.78) | 32.13 (30.78 - 33.48) |
| Missouri | 15.62 (15.37 - 15.87) | 11 (10.46 - 11.53) |
| Montana | 7.28 (6.87 - 7.69) | 8.17 (7.12 - 9.22) |
| Nebraska | 8.66 (8.32 - 9) | 8.85 (7.98 - 9.71) |
| Nevada | 5.09 (4.86 - 5.32) | 6.28 (5.7 - 6.87) |
| New Hampshire | 10.51 (10.07 - 10.95) | 6.36 (5.55 - 7.17) |
| New Jersey | 12.68 (12.49 - 12.86) | 7.51 (7.16 - 7.87) |
| New Mexico | 10.35 (9.99 - 10.71) | 9.99 (9.11 - 10.86) |
| New York | 10.42 (10.31 - 10.53) | 7.17 (6.93 - 7.4) |
| North Carolina | 14.75 (14.55 - 14.95) | 12.95 (12.5 - 13.39) |
| North Dakota | 13.66 (12.98 - 14.34) | 9.74 (8.3 - 11.19) |
| Ohio | 17.82 (17.63 - 18.01) | 14.11 (13.67 - 14.54) |
| Oklahoma | 13.51 (13.21 - 13.81) | 11.14 (10.45 - 11.83) |
| Oregon | 11.66 (11.4 - 11.93) | 11.37 (10.73 - 12.01) |
| Pennsylvania | 14.24 (14.09 - 14.4) | 10.62 (10.27 - 10.96) |
| Rhode Island | 17.94 (17.33 - 18.55) | 10.12 (8.95 - 11.28) |
| South Carolina | 15.25 (14.97 - 15.54) | 14.96 (14.29 - 15.62) |
| South Dakota | 17.52 (16.81 - 18.22) | 21.92 (19.91 - 23.94) |
| Tennessee | 18.21 (17.94 - 18.47) | 17.52 (16.88 - 18.15) |
| Texas | 16.24 (16.09 - 16.38) | 13.49 (13.19 - 13.79) |
| Utah | 9.3 (8.96 - 9.65) | 9.1 (8.32 - 9.89) |
| Vermont | 13.07 (12.38 - 13.75) | 8.51 (7.16 - 9.87) |
| Virginia | 11.1 (10.9 - 11.29) | 10.63 (10.18 - 11.08) |
| Washington | 12.48 (12.26 - 12.7) | 11.2 (10.7 - 11.69) |
| West Virginia | 18.98 (18.51 - 19.44) | 16.6 (15.46 - 17.75) |
| Wisconsin | 13.56 (13.32 - 13.8) | 13.2 (12.61 - 13.78) |
| Wyoming | 12.96 (12.18 - 13.74) | 12.99 (11.08 - 14.9) |
| Total | 12.97 (12.94 - 13) | 11.03 (10.96 - 11.1) |

**Table S6:** DM and MI related Age Adjusted Mortality Rates per 100,000 stratified by Census Region in the United States; 1999-2023.

| **Age Adjusted Mortality Rate per 100,000 (95% CI)** | | | | |
| --- | --- | --- | --- | --- |
| **Year** | **Northeast** | **Midwest** | **South** | **West** |
| 1999 | 18.88 (18.44 - 19.32) | 20.79 (20.35 - 21.23) | 19.07 (18.73 - 19.41) | 16.82 (16.39 - 17.25) |
| 2000 | 18.11 (17.68 - 18.54) | 19.76 (19.34 - 20.19) | 19.45 (19.11 - 19.79) | 16.19 (15.77 - 16.61) |
| 2001 | 17.13 (16.71 - 17.54) | 18.86 (18.45 - 19.27) | 18.31 (17.98 - 18.64) | 15.75 (15.34 - 16.15) |
| 2002 | 16.2 (15.79 - 16.6) | 18.23 (17.83 - 18.63) | 18.4 (18.07 - 18.73) | 16.17 (15.77 - 16.58) |
| 2003 | 15.58 (15.18 - 15.97) | 17.36 (16.97 - 17.76) | 17.59 (17.27 - 17.91) | 15.4 (15.01 - 15.79) |
| 2004 | 14.62 (14.24 - 15) | 16.11 (15.73 - 16.48) | 16.28 (15.98 - 16.58) | 14.6 (14.23 - 14.98) |
| 2005 | 13.99 (13.62 - 14.36) | 16.08 (15.71 - 16.45) | 15.63 (15.34 - 15.92) | 14.13 (13.76 - 14.5) |
| 2006 | 12.91 (12.56 - 13.26) | 15.05 (14.69 - 15.4) | 14.8 (14.52 - 15.09) | 13.44 (13.09 - 13.79) |
| 2007 | 12.27 (11.92 - 12.61) | 14.01 (13.67 - 14.35) | 13.72 (13.45 - 13.98) | 12.63 (12.29 - 12.97) |
| 2008 | 11.61 (11.28 - 11.94) | 14.05 (13.71 - 14.39) | 13.63 (13.36 - 13.89) | 12.5 (12.16 - 12.83) |
| 2009 | 10.52 (10.2 - 10.83) | 13.06 (12.73 - 13.38) | 13.19 (12.93 - 13.45) | 11.36 (11.05 - 11.67) |
| 2010 | 10.36 (10.05 - 10.67) | 12.44 (12.13 - 12.76) | 12.61 (12.36 - 12.86) | 11.6 (11.29 - 11.91) |
| 2011 | 9.99 (9.69 - 10.29) | 12.89 (12.57 - 13.21) | 12.25 (12.01 - 12.5) | 10.84 (10.54 - 11.14) |
| 2012 | 9.62 (9.32 - 9.91) | 11.97 (11.67 - 12.28) | 11.88 (11.65 - 12.12) | 10.68 (10.38 - 10.97) |
| 2013 | 9.33 (9.04 - 9.62) | 12.14 (11.83 - 12.44) | 11.43 (11.2 - 11.65) | 10.6 (10.31 - 10.89) |
| 2014 | 8.95 (8.67 - 9.24) | 11.67 (11.38 - 11.97) | 11.16 (10.93 - 11.38) | 10.21 (9.93 - 10.49) |
| 2015 | 8.6 (8.33 - 8.87) | 11.17 (10.89 - 11.46) | 11.14 (10.92 - 11.36) | 10.41 (10.13 - 10.68) |
| 2016 | 8.34 (8.07 - 8.61) | 10.83 (10.54 - 11.11) | 11.14 (10.93 - 11.36) | 10.32 (10.05 - 10.6) |
| 2017 | 8.02 (7.76 - 8.28) | 11.03 (10.75 - 11.31) | 11.16 (10.95 - 11.38) | 10.25 (9.98 - 10.51) |
| 2018 | 8.02 (7.77 - 8.28) | 10.81 (10.54 - 11.09) | 11.12 (10.91 - 11.34) | 9.84 (9.59 - 10.1) |
| 2019 | 7.84 (7.58 - 8.09) | 10.42 (10.15 - 10.69) | 11.21 (11 - 11.42) | 9.76 (9.5 - 10.01) |
| 2020 | 9.31 (9.04 - 9.59) | 11.64 (11.36 - 11.92) | 13.39 (13.16 - 13.61) | 11.35 (11.08 - 11.62) |
| 2021 | 8.56 (8.3 - 8.82) | 12.5 (12.2 - 12.79) | 14.28 (14.04 - 14.52) | 11.71 (11.43 - 11.99) |
| 2022 | 7.9 (7.66 - 8.15) | 10.91 (10.64 - 11.19) | 12.77 (12.55 - 12.99) | 10.89 (10.63 - 11.16) |
| 2023 | 6.89 (6.66 - 7.12) | 9.51 (9.26 - 9.77) | 11.5 (11.3 - 11.71) | 9.29 (9.05 - 9.53) |

**Table S7:** DM and MI related Age Adjusted Mortality Rates per 100,000 stratified by Urban-Rural status in the United States; 1999-2023.

| **Age Adjusted Mortality Rate per 100,000 (95% CI)** | | |
| --- | --- | --- |
| **Year** | **Urban** | **Rural** |
| 1999 | 21.78 (21.28 - 22.28) | 18.39 (18.16 - 18.61) |
| 2000 | 21.71 (21.21 - 22.21) | 17.9 (17.68 - 18.12) |
| 2001 | 20.69 (20.21 - 21.18) | 17.01 (16.8 - 17.22) |
| 2002 | 21.23 (20.74 - 21.72) | 16.63 (16.42 - 16.83) |
| 2003 | 20.48 (20 - 20.96) | 15.88 (15.68 - 16.08) |
| 2004 | 19.08 (18.62 - 19.54) | 14.82 (14.63 - 15.01) |
| 2005 | 18.75 (18.29 - 19.2) | 14.33 (14.15 - 14.52) |
| 2006 | 17.5 (17.07 - 17.93) | 13.52 (13.34 - 13.7) |
| 2007 | 16.97 (16.55 - 17.4) | 12.46 (12.29 - 12.63) |
| 2008 | 17.37 (16.94 - 17.79) | 12.18 (12.02 - 12.35) |
| 2009 | 16.75 (16.34 - 17.17) | 11.31 (11.16 - 11.47) |
| 2010 | 15.89 (15.49 - 16.3) | 11.12 (10.96 - 11.27) |
| 2011 | 16.35 (15.94 - 16.76) | 10.7 (10.55 - 10.85) |
| 2012 | 15.49 (15.1 - 15.89) | 10.35 (10.2 - 10.5) |
| 2013 | 15.42 (15.03 - 15.81) | 10.11 (9.97 - 10.25) |
| 2014 | 15.26 (14.87 - 15.65) | 9.73 (9.59 - 9.87) |
| 2015 | 15.36 (14.97 - 15.75) | 9.56 (9.43 - 9.7) |
| 2016 | 14.9 (14.52 - 15.28) | 9.51 (9.37 - 9.64) |
| 2017 | 15.56 (15.17 - 15.95) | 9.37 (9.24 - 9.51) |
| 2018 | 15.52 (15.13 - 15.9) | 9.16 (9.03 - 9.29) |
| 2019 | 15.54 (15.15 - 15.92) | 9.06 (8.93 - 9.19) |
| 2020 | 18.04 (17.62 - 18.45) | 10.69 (10.55 - 10.83) |

**Table S8:** DM and MI related Crude Mortality Rates per 100,000 stratified by 10-year age groups in the United States; 1999-2023.

| **Crude Mortality Rate per 100,000 (95% CI)** | | | | | | | |
| --- | --- | --- | --- | --- | --- | --- | --- |
| **Year** | **25-34 years** | **35-44 years** | **45-54 years** | **55-64 years** | **65-74 years** | **75-84 years** | **85+ years** |
| 1999 | 0.22 (0.18 - 0.27) | 1.18 (1.08 - 1.28) | 6.25 (5.99 - 6.51) | 22.15 (21.56 - 22.75) | 51.01 (49.98 - 52.04) | 89.19 (87.51 - 90.86) | 123.52 (120.14 - 126.9) |
| 2000 | 0.21 (0.16 - 0.26) | 1.21 (1.11 - 1.31) | 6.1 (5.85 - 6.35) | 21.11 (20.53 - 21.69) | 49.3 (48.29 - 50.32) | 87.52 (85.87 - 89.17) | 126.36 (122.97 - 129.74) |
| 2001 | 0.16 (0.12 - 0.2) | 1.17 (1.07 - 1.27) | 5.89 (5.65 - 6.13) | 19.53 (18.99 - 20.08) | 46.89 (45.9 - 47.88) | 82.92 (81.33 - 84.51) | 122.92 (119.61 - 126.23) |
| 2002 | 0.19 (0.15 - 0.24) | 1.26 (1.16 - 1.37) | 5.89 (5.66 - 6.13) | 19.57 (19.04 - 20.1) | 45.09 (44.12 - 46.06) | 81.3 (79.74 - 82.87) | 124.63 (121.32 - 127.94) |
| 2003 | 0.21 (0.16 - 0.26) | 1.26 (1.16 - 1.37) | 5.72 (5.49 - 5.95) | 18.9 (18.4 - 19.41) | 43.25 (42.3 - 44.2) | 77.34 (75.82 - 78.86) | 116.86 (113.69 - 120.03) |
| 2004 | 0.19 (0.15 - 0.24) | 1.17 (1.07 - 1.27) | 5.49 (5.27 - 5.72) | 17.51 (17.03 - 17.99) | 40.16 (39.25 - 41.07) | 71.43 (69.98 - 72.89) | 110.89 (107.83 - 113.95) |
| 2005 | 0.15 (0.11 - 0.19) | 1.22 (1.11 - 1.32) | 5.04 (4.82 - 5.25) | 16.93 (16.47 - 17.4) | 38.31 (37.43 - 39.2) | 70.38 (68.94 - 71.82) | 110.52 (107.51 - 113.53) |
| 2006 | 0.18 (0.14 - 0.23) | 1.1 (1 - 1.2) | 5.1 (4.89 - 5.31) | 16 (15.56 - 16.44) | 35.47 (34.63 - 36.32) | 66.32 (64.93 - 67.72) | 101.75 (98.91 - 104.58) |
| 2007 | 0.15 (0.12 - 0.19) | 1.18 (1.07 - 1.28) | 4.83 (4.62 - 5.03) | 14.69 (14.28 - 15.1) | 33.14 (32.34 - 33.95) | 61.02 (59.68 - 62.36) | 97.55 (94.82 - 100.28) |
| 2008 | 0.15 (0.11 - 0.19) | 1.05 (0.96 - 1.15) | 4.8 (4.59 - 5) | 14.81 (14.41 - 15.22) | 32.86 (32.07 - 33.64) | 59.67 (58.35 - 61) | 96.4 (93.73 - 99.07) |
| 2009 | 0.15 (0.12 - 0.2) | 1.09 (0.99 - 1.19) | 4.59 (4.39 - 4.79) | 13.85 (13.46 - 14.24) | 30.48 (29.73 - 31.22) | 56.06 (54.77 - 57.34) | 88.93 (86.4 - 91.45) |
| 2010 | 0.18 (0.14 - 0.22) | 1 (0.9 - 1.09) | 4.53 (4.34 - 4.73) | 13.75 (13.37 - 14.13) | 29.56 (28.83 - 30.28) | 54.29 (53.03 - 55.55) | 85.34 (82.9 - 87.78) |
| 2011 | 0.17 (0.13 - 0.21) | 1.13 (1.03 - 1.24) | 4.6 (4.4 - 4.79) | 13.48 (13.11 - 13.85) | 28.83 (28.13 - 29.53) | 52.87 (51.63 - 54.11) | 81.49 (79.15 - 83.82) |
| 2012 | 0.17 (0.13 - 0.21) | 1.04 (0.94 - 1.14) | 4.38 (4.19 - 4.58) | 12.89 (12.53 - 13.24) | 27.67 (27.01 - 28.34) | 50.46 (49.26 - 51.67) | 80.92 (78.62 - 83.22) |
| 2013 | 0.16 (0.12 - 0.2) | 1.03 (0.93 - 1.13) | 4.54 (4.34 - 4.73) | 12.61 (12.26 - 12.96) | 27.32 (26.67 - 27.96) | 49.05 (47.86 - 50.23) | 78.07 (75.84 - 80.3) |
| 2014 | 0.14 (0.11 - 0.19) | 1.1 (1 - 1.2) | 4.46 (4.26 - 4.66) | 12.6 (12.25 - 12.94) | 26.41 (25.79 - 27.03) | 47.59 (46.43 - 48.74) | 71.48 (69.37 - 73.59) |
| 2015 | 0.12 (0.09 - 0.16) | 0.98 (0.88 - 1.07) | 4.33 (4.13 - 4.52) | 12.77 (12.42 - 13.11) | 26.51 (25.91 - 27.12) | 46.12 (44.99 - 47.25) | 71.18 (69.09 - 73.26) |
| 2016 | 0.16 (0.12 - 0.2) | 1 (0.9 - 1.1) | 4.53 (4.33 - 4.74) | 12.51 (12.16 - 12.85) | 25.71 (25.12 - 26.29) | 45.86 (44.74 - 46.97) | 69.31 (67.26 - 71.35) |
| 2017 | 0.15 (0.11 - 0.19) | 0.98 (0.88 - 1.07) | 4.52 (4.32 - 4.73) | 12.24 (11.9 - 12.57) | 25.8 (25.22 - 26.37) | 46.44 (45.34 - 47.54) | 68.25 (66.24 - 70.27) |
| 2018 | 0.14 (0.11 - 0.18) | 0.98 (0.88 - 1.07) | 4.46 (4.26 - 4.67) | 12.34 (12 - 12.67) | 25.93 (25.36 - 26.51) | 44.33 (43.28 - 45.38) | 67.29 (65.31 - 69.28) |
| 2019 | 0.15 (0.12 - 0.19) | 1.03 (0.93 - 1.13) | 4.39 (4.18 - 4.59) | 12.01 (11.68 - 12.34) | 25.13 (24.58 - 25.69) | 45.13 (44.09 - 46.17) | 65.62 (63.66 - 67.57) |
| 2020 | 0.2 (0.16 - 0.24) | 1.27 (1.16 - 1.37) | 5.27 (5.04 - 5.49) | 14.42 (14.06 - 14.78) | 29.55 (28.96 - 30.14) | 51.56 (50.47 - 52.66) | 75.96 (73.87 - 78.06) |
| 2021 | 0.19 (0.15 - 0.24) | 1.27 (1.16 - 1.38) | 5.52 (5.29 - 5.74) | 14.92 (14.55 - 15.28) | 29.36 (28.78 - 29.94) | 54.22 (53.09 - 55.35) | 82.9 (80.59 - 85.21) |
| 2022 | 0.16 (0.13 - 0.21) | 1.18 (1.07 - 1.28) | 4.95 (4.73 - 5.16) | 13.24 (12.89 - 13.58) | 26.81 (26.26 - 27.36) | 48.58 (47.55 - 49.61) | 75.15 (73.04 - 77.26) |
| 2023 | 0.13 (0.1 - 0.16) | 1.02 (0.92 - 1.11) | 4.47 (4.26 - 4.68) | 11.57 (11.25 - 11.9) | 23.92 (23.4 - 24.44) | 42.6 (41.63 - 43.57) | 64.74 (62.78 - 66.7) |
